# Measuring Alphavirus Fidelity Using Non-Infectious Virus Particles

**DOI:** 10.3390/v12050546

**Published:** 2020-05-15

**Authors:** Edward I. Patterson, Kamil Khanipov, Daniele M. Swetnam, Samantha Walsdorf, Tiffany F. Kautz, Saravanan Thangamani, Yuriy Fofanov, Naomi L. Forrester

**Affiliations:** 1 Department of Pathology, University of Texas Medical Branch, Galveston, TX 77555, USA; samleigh@comcast.net (S.W.); thangams@upstate.edu (S.T.); n.forrester-soto@keele.ac.uk (N.L.F.); 2 Centre for Neglected Tropical Diseases, Departments of Vector Biology and Tropical Disease Biology, Liverpool School of Tropical Medicine, Pembroke Place, Liverpool L3 5QA, UK; 3 Department of Pharmacology and Toxicology, Sealy Center for Structural Biology and Molecular Biophysics, University of Texas Medical Branch, Galveston, TX 77555, USA; kakhanip@utmb.edu (K.K.); yufofano@utmb.edu (Y.F.); 4 Department of Pathology, Microbiology, and Immunology, School of Veterinary Medicine, University of California, Davis, Davis, CA 95616, USA; dmswetnam@ucdavis.edu; 5 Department of Microbiology and Immunology, University of Texas Medical Branch, Galveston, TX 77555, USA; kautz@uthscsa.edu

**Keywords:** alphavirus, arbovirus, fidelity mutants, mutation frequency

## Abstract

Mutations are incorporated into the genomes of RNA viruses at an optimal frequency and altering this precise frequency has been proposed as a strategy to create live-attenuated vaccines. However, determining the effect of specific mutations that alter fidelity has been difficult because of the rapid selection of the virus population during replication. By deleting residues of the structural polyprotein PE2 cleavage site, E3Δ56-59, in Venezuelan equine encephalitis virus (VEEV) TC-83 vaccine strain, non-infectious virus particles were used to assess the effect of single mutations on mutation frequency without the interference of selection that results from multiple replication cycles. Next-generation sequencing analysis revealed a significantly lower frequency of transversion mutations and overall mutation frequency for the fidelity mutants compared to VEEV TC-83 E3Δ56-59. We demonstrate that deletion of the PE2 cleavage site halts virus infection while making the virus particles available for downstream sequencing. The conservation of the site will allow the evaluation of suspected fidelity mutants across alphaviruses of medical importance.

## 1. Introduction

RNA viruses are ubiquitous in nature. The success of RNA viruses is partially due to their strategy of generating a large number of mutations to create a cloud of closely related mutants, which is commonly termed a viral “quasispecies”. The viruses that comprise the quasispecies are believed to act co-operatively together to create a successful infection both within and outside the cell [1,2,3]. This replication strategy is not without its drawbacks as RNA viruses have evolved to exist where the mutation rate is high enough to be beneficial, while the number of deleterious mutations remain below levels that would otherwise result in error catastrophe, the state where excessive mutations render proteins non-functional and can potentially lead to viral extinction. If this mutation rate is altered, even slightly, the resulting virus population commonly exhibits attenuation, especially in vivo [3,4,5,6,7,8,9,10,11,12]. Thus, an optimized mutation rate is essential for RNA virus transmission and infection.

This inherent property of RNA viruses has allowed the generation of virus mutants that alter the balance of the mutational spectrum, resulting in viruses with either increased (low-fidelity) or decreased (high-fidelity) mutation rates. These viruses have been generated either by replicating the viruses under selective pressures by the use of nucleoside analogues [13,14,15,16] or by mutating the RNA-dependent RNA polymerase (RdRp) using known mutations from related viruses [12,17]. These altered fidelity viruses have allowed us and other researchers to identify the effects of manipulating the RNA virus mutation rate, with every altered virus that has been generated showing some evidence of attenuation [7,12,18,19,20,21,22]. Conversely, as recently detailed, there have been numerous examples of issues with reproducibility and identifying fidelity mutants consistently [23]. While additional studies on the mechanistic effects of the mutations are necessary, part of the inconsistencies may be the result of different methods used to generate altered fidelity viruses coupled with the different methods used to produce virus stocks.

**Figure 1:**
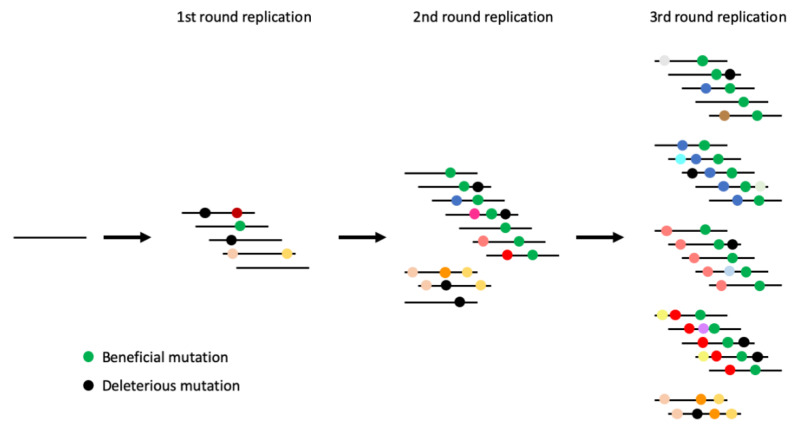
Schematic of mutation accumulation across several rounds of virus replication. Lines represent the virus genome and colored dots represent mutations. Measuring mutation frequency after a single round of replication provides the most accurate representation of the fidelity of the sequence of interest. Collecting samples from subsequent rounds of replication will inadvertently measure the effect of secondary mutants accumulated during replication.

Fidelity mutant viruses have the potential to be effective live-attenuated vaccines, as they result in attenuation of the virus and can increase immunogenicity [12,24]. However, the inconsistency of the results associated with fidelity mutants hampers the development of these viruses as a promising vaccine strategy. One major issue is that the amount of diversity generated by the virus can be masked by variation generated by multiple replication cycles of the virus. Additional replication cycles may instead measure the varying replication rates of randomly generated mutants, rather than variation of the original fidelity mutant. To counter this, we developed an assay that allowed us to measure the fidelity of a virus after just one replication cycle, thus bypassing the inherent bias of multiple replication cycles (Figure 1). We found that the approach described here helps control for some of the inconsistencies associated with virus fidelity mutants and provides a novel framework for a controlled measure of virus replicative fidelity. 

## 2. Materials and Methods

### 2.1. Cell Culture and Viruses

Vero E6 (African green monkey kidney; CRL-1586) cells were sourced from the American Type Cell Culture Collection (ATCC; Bethesda, MD, USA). Vero cell cultures were maintained in Dulbecco’s minimal essential medium (DMEM) containing 10% fetal bovine serum (FBS) and 0.05 mg/mL of gentamycin at 37 °C with 5% CO_2_. 

**Figure 2:**
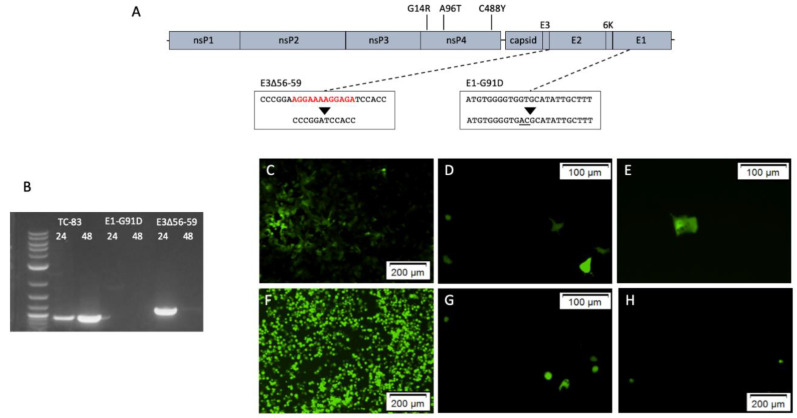
Mutations added to Venezuelan equine encephalitis virus (VEEV) TC-83 genome to inhibit infectivity. Schematic of the VEEV genome showing the location of the E3Δ56-59 and E1-G91D mutations and mutations suspected of altering fidelity (**A**). RT-PCR of media collected from VEEV TC-83, VEEV TC-83 E3Δ56-59 and VEEV TC-83 E1-G91D at 24 and 48 h post-electroporation (**B**). Fluorescent microscopy of Vero cells transfected with VEEV TC-83 GFP (**C**,**F**), VEEV TC-83 E3Δ56-59 GFP (**D**,**G**) and VEEV TC-83 E1-G91D GFP (**E**,**H**). Panels C-E are 24 h post-transfection, F-H are 48 h post-transfection.

Infectious clones of Venezuelan equine encephalitis virus (VEEV) TC-83 were used to create the PE2 cleavage site deletion mutants [25,26,27]. The backbone infectious clones encoded VEEV TC-83 strain, subclones for fidelity mutants (nsP4-G14R, nsP4-A96T, and nsP4-C488Y) [12] and VEEV TC-83 GFP (provided by Dr. Weaver from UTMB) (Figure 2A). The PE2 cleavage site residues (E3Δ56-59) were deleted by In-Fusion HD cloning (Takara Bio; Mountain View, CA, USA) using the forward primer 5′-CCCCGGATCCACCGAGGAGCTGTTTAATGAG-3′ and reverse primer 5′-TCGGTGGATCCGGGGCACTTAACAGCTG-3′ according to the manufacturer’s instructions. Deletions were confirmed by Sanger sequencing and the sequences of the entire plasmids were confirmed by Illumina sequencing. Library preparation on gDNA was performed using Illumina XT DNA Library kit. Paired-end sequencing was performed on an Illumina MiSeq using 500 cycle V2 cartridge. Infectious clones were transcribed by in-vitro transcription and rescued by electroporation in Vero cells, in triplicate, as previously described [28]. 

The envelope mutation, E1-G91D, was also cloned into the infectious clones for VEEV TC-83 and TC-83 GFP [29,30]. The E1-G91D mutation was made using with the In-Fusion HD cloning kit with the forward primer 5′-TGGGGTGACGCATATTGCTTTTGC-3′ and reverse primer 5′- ATATGCGTCACCCCACATGAACG-3′ according to the manufacturer’s instructions. The mutation in the plasmids was confirmed by Sanger sequencing and the entire plasmids were sequenced by Illumina sequencing as above.

### 2.2. Virus Detection and Replication Assays

Cytopathic effect (CPE) assays were performed in 12-well plates and incubated for 72 h to detect replicating virus as previously described [31]. Media was removed from wells and replaced with fresh media every 24 h. RNA was extracted from media used to rescue virus with a QIAamp RNA mini kit (Qiagen; Germantown, MD, USA) according to the manufacturer’s instructions. RT-PCR was performed with a Qiagen one-step RT-PCR kit according to the manufacturer’s instructions with primers 9885V (5′-TCTGGATTCAATTGCTGATCC-3′) and 10749C (5′-AGTTTTCGGCGCGAATGG-3′) for VEEV TC-83 and TC-83 E1-G91D, or 7701V (5′-ACCTGACGTTCAAGCAACG-3′) and 8763C (5′-GTTCCGTGCATGTCATACC-3′) for TC-83 E3Δ56-59.

Virus clones containing GFP insertions were transfected with Lipofectamine 2000 reagent (Invitrogen; Waltham, MA, USA) into Vero cell cultures in a 12-well plate according to the manufacturer’s instructions. After 4 h, media was removed and replaced with fresh media containing 2% FBS. Fluorescent microscopy was used to observe the replication of viruses expressing GFP.

### 2.3. Analysis of Mutation Frequency

In vitro transcripts were used to electroporate cells in triplicate and seeded in 10 mL of media in T75 flasks. Media from each flask was harvested after 24 h, clarified by centrifugation, and PEG precipitated to concentrate virus. Concentrated virus was resuspended in PBS and stored at −80 °C until further processing. RNA was extracted from concentrated samples with a QIAamp RNA mini kit (Qiagen) according to the manufacturer’s instructions. Library preparation was performed as previously described [12].

### 2.4. Analysis Counting All Variants (Analysis A)

Sequence analysis was performed as previously described [12]. The quality for each sample/dataset was assessed using FASTQC [32]. The reads were filtered to exclude reads with unknown characters and low (<10) quality scores. Additionally, the first 16 bases of each read were trimmed due to nucleotide bias. The analysis was performed using VEEV strain TC-83, complete genome (GenBank: L01443.1), with the deleted nucleotides removed [33]. To analyze the variant hotspots, each sample was run through a rare-variant pipeline (available upon request). The pipeline first exhaustively maps each read to the reference VEEV genome without allowing mismatches, then unmapped reads are re-mapped with one mismatch and added to the map. The 34-base long reads used in the analyses were confirmed as viral sequences and not host sequences by comparison of the longest subsequences shared (up to one mismatch) between viral and host genomes [34]. Diversity per position in the viral genome was calculated by taking a ratio of the reads mapped to non-reference alleles and total number of reads mapped at that position. Positions in which the number of reads mapped with non-reference alleles was higher than reads mapped to reference alleles or coverage was below 100 were excluded from diversity calculations (Table S1). Mutation frequency per sample was calculated by summing per-position diversities and normalizing by the number of positions for which diversity was calculated. Positions with non-zero mutation frequency were considered to be variant positions.

### 2.5. Analysis with Downsampled Dataset (Analysis B)

Reads were aligned to the reference genome based on the VEEV strain TC-83, complete genome (GenBank: L01443.1), with the deleted nucleotides removed using the very sensitive local setting in Bowtie2 version 2.3.2 [35]. Bam files were sorted, indexed, processed using SAMTools Version 1.4.1 (http://www.htslib.org/) and Picard 2.20.5 (https://broadinstitute.github.io/picard/). Files were downsampled to a mean coverage of 2000 reads/position. Intra-host variation was investigated using the DeepSNV library within R. Low-quality reads (q < 10) were excluded from further analysis. Mutation frequency was calculated as (N-n/N) where N is equal to the total number of reads and n is equal to the number of reads matching the dominant genotype.

### 2.6. Statistics

All statistical analyses were performed using GraphPad Prism. Overall diversity was compared with one-way ANOVA and Tukey’s multiple comparisons test.

## 3. Results 

### 3.1. Virus Replication

The PE2 cleavage site deletion, E3Δ56-59, and envelope mutation, E1-G91D, were successfully cloned into separate VEEV TC-83 plasmids, and viral RNA was electroporated into cells. To determine whether the rescued viruses were infectious, a CPE assay was performed on media collected at 24 h and 48 h post-electroporation from unmutated VEEV TC-83 control, TC-83 E3Δ56-59, and TC-83 E1-G91D. Wells inoculated with harvested media from TC-83 E3Δ56-59 and TC-83 E1-G91D electroporations were negative for CPE, while the TC-83 control wells were positive for CPE. These results indicate that the media collected from the TC-83 E3Δ56-59 and TC-83 E1-G91D electroporations is non-infectious.

As the CPE assay alone does not indicate virus presence or replication, RT-PCR was performed on RNA extracted from the harvested media. The correct bands were seen with samples from the TC-83 control as well as 24 h post-electroporation with TC-83 E3Δ56-59, and a slight band at 48 h post-electroporation with TC-83 E3Δ56-59, corresponding to a larger release of non-infectious virus particles at 24 h post-electroporation. Sequencing the RT-PCR product confirmed the deletion in TC-83 E3Δ56-59 at 24 h post-electroporation. No bands were seen for TC-83 E1-G91D samples (Figure 2B). These results indicate that TC-83 E3Δ56-59 has replicated following electroporation and is present in the media, while TC-83 E1-G91D is not present in the media.

To confirm these results and determine if TC-83 E1-G91D is replicating, TC-83 sequences containing a GFP reporter were transfected into cells. Fluorescent microscopy with control TC-83 revealed replication in adjacent cells, with increased GFP expression between 24 to 48 h post-transfection (Figure 2C,F). Both TC-83 E3Δ56-59 and TC-83 E1-G91D displayed GFP expression in isolated cells, without the spread of virus into adjacent cells (Figure 2D,E,G,H). This indicates that the viral genome is replicating, but infectious virus particles are not produced. 

### 3.2. Overall Sequence Diversity

**Figure 3:**
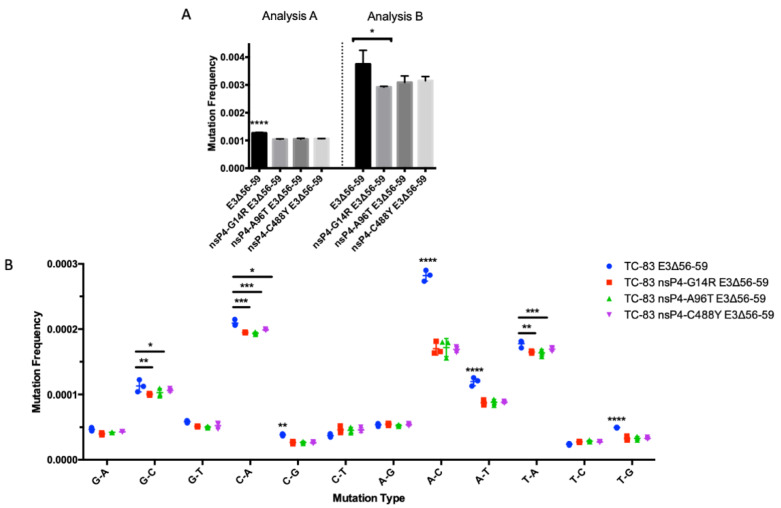
Mutation frequency of VEEV TC-83 E3Δ56-59 mutants. Overall mutation frequency of VEEV TC-83 E3Δ56-59 and mutants with nsP4 mutations that alter fidelity using two methods for analysis (**A**). Frequency of specific mutation types of VEEV TC-83 E3Δ56-59 and mutants with nsP4 mutations that alter fidelity with Analysis A (**B**). Mutation frequency was compared with one-way ANOVA and Tukey’s multiple comparisons test. *p* < 0.05 is represented by *, *p* < 0.01 is represented by **, *p* < 0.001 is represented by *** and *p* < 0.0001 is represented by ****.

The E3Δ56-59 deletion was used to aid measurement of the sequence diversity of VEEV isolates by preventing infectious cycles in TC-83 and mutants suspected of altering fidelity. Media from control and fidelity mutants was collected at 24 h post-electroporation in triplicate. The mutation frequency of TC-83 E3Δ56-59 was significantly higher than all the putative fidelity mutants, TC-83 E3Δ56-59 nsP4-G14R, TC-83 E3Δ56-59 nsP4-A96T and TC-83 E3Δ56-59 nsP4-C488Y using Analysis A (Figure 3A). For Analysis B, the mutation frequency of TC-83 E3Δ56-59 was significantly higher than TC-83 E3Δ56-59 nsP4-G14R, but not significantly different to other mutants. There was no significant difference in the mutation frequency among fidelity mutants with either analysis method. The mutation frequencies observed with Analysis A were approximately three times lower than those observed with Analysis B for each condition. Several types of mutations were significantly more frequent in TC-83 E3Δ56-59 compared to the fidelity mutants (Figure 3B). All of the significantly different mutation types were transversions: G-C, C-A, C-G, A-C, A-T, T-A and T-G. Fidelity mutants did not significantly differ among mutation types. 

### 3.3. Sequence Diversity across the Genome

**Figure 4:**
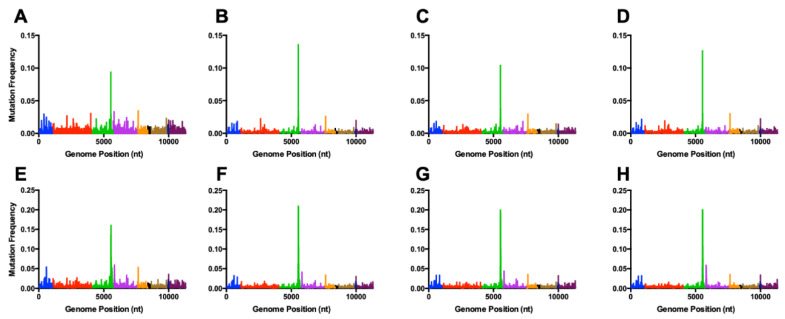
Mutation frequency of VEEV TC-83 E3Δ56-59 mutants at coding regions across the genome. VEEV TC-83 E3Δ56-59 (**A**,**E**), VEEV TC-83 E3Δ56-59 nsP4-G14R (**B**,**F**), VEEV TC-83 E3Δ56-59 nsP4-A96T (**C**,**G**), and VEEV TC-83 E3Δ56-59 nsP4-C488Y (**D**,**H**). Panels A-D have been analyzed using Analysis A. Panels E-H have been analyzed using Analysis B. Graphs show a representative of each specific mutant. Blue = nsP1, red = nsP2, green = nsP3, purple = nsP4, orange = capsid, black = E3, gold = E2, navy blue = 6k, maroon = E1.

To see if mutations accumulated at specific sequence locations, mutation frequency was viewed at all points across the genome. All virus isolates exhibited similar profiles for mutation frequency along the genome, with a large peak of diversity seen at the 3′ end of the nsP3 gene (Figure 4; Figure S1). Similar trends for mutation frequency along the genome were seen when comparing samples from Analysis A and Analysis B. Several of the most frequent mutations are found across many samples, with C5540A (nsP3), C5555A (nsP3) and A7653C (capsid) found at high frequencies in all samples (Table 1; Table S2). 

## 4. Discussion

Replication of RNA viruses occurs within an optimal range of mutation frequency. Disturbing the rate that mutations are incorporated into the virus genome is detrimental to the virus and leads to attenuation [8,36,37]. This is of particular interest because altering fidelity has been proposed as an effective method for generating live-attenuated vaccines with increased stability. While fidelity mutants have been reported for many viruses, interpretation of the effects of altered fidelity vary, especially among the studied alphavirus and poliovirus candidates [5,7,8,9,12,23,37,38]. Optimizing an assay to evaluate mutation rate with reproducible results will be a priority for vaccine development of fidelity mutants. This is especially challenging for alphaviruses, as the RdRp is unstable [23], so methods that require isolation of the RdRp to measure nucleotide incorporation rates are not viable.

Ideally, only a single round of infection should be measured to determine the effect of a specific mutation suspected to alter fidelity. This would eliminate biased data from fitness imparted by random mutations that are selected during sequential infection of cells. We created a method to evaluate fidelity mutants by inserting previously characterized mutations that block either virus binding to or virus release from the host cell, E3Δ56-59 [25,26,27] and E1-G91D [29,30] into the structural proteins of the VEEV vaccine strain, TC-83. The media harvested from both TC-83 E3Δ56-59 and TC-83 E1-G91D electroporation were demonstrated to be non-infectious, while replication of the viral genomes was verified to have occurred following transfection. However, TC-83 E3Δ56-59 provided a distinct advantage for downstream next-generation sequencing analysis by having virus genomes accessible in the media. 

These results are consistent with previous reports from alphaviruses containing either the E3Δ56-59 or E1-G91D mutations. The E1-G91D mutation interferes with E1 trimer formation, which is necessary for viral assembly and release from the cell [29,30]. Deletion of E3Δ56-59 eliminates the PE2 cleavage site and results in an unprocessed E2-E3 envelope protein. For Sindbis virus, Semliki Forest virus and VEEV, the uncleaved E2-E3 does not inhibit the formation of virus particles or budding from the cell. However, the resulting viruses are blocked at early points of infection, notably at binding to host cells [25,39]. Although our experiments did not isolate viral protein, the purification of virus RNA from the media suggests that intact virions were produced from the electroporation, as was seen with other alphaviruses with the E3Δ56-59 deletion, including VEEV [25,26,27,39]. These characteristics meet the criteria we outlined for measuring the effects of a specific mutation on virus fidelity.

To use this system of a budding, non-infectious virus to measure mutation frequency in the viral genome, we deleted E3Δ56-59 in VEEV TC-83 plasmids containing mutations suspected to alter fidelity [12]. The G14R and A96T mutations were previously identified following 19 and 23 passages of TC-83 with 5′fluorouracil [12], while C488Y is the corresponding mutation to C483Y identified in the high-fidelity chikungunya virus (CHIKV) [7]. The samples in this study containing these RdRp mutations were found to produce significantly lower mutation frequencies, characteristic of high-fidelity virus, as well as having lower rates of transversion mutations. 

Previous studies with TC-83 did not evaluate the mutation frequency of the fidelity mutants individually. However, Kautz et al. [12] concluded that an isolate containing four mutations, 4×, did not show significantly different mutation frequency to TC-83, while a 3× mutant, containing nsP4-G14R, E37G and A96T exhibited a low fidelity phenotype in Vero cells. Unlike the single RdRp mutants measured in this paper, the 3× and 4× isolates had significantly fewer U-C transition mutations, suggesting that inserting these RdRp mutations into the same TC-83 genome changed the types of mutations that are generated by the RdRp, which in turn lowered the overall fidelity. The high-fidelity C483Y mutation has only been evaluated in isolation with CHIKV but was recently recharacterized with next-generation sequencing across the entire genome [38]. After comparing mutation frequency across the genome, Riemersma et al. [38] saw no significant difference between wild-type CHIKV and CHIKV C483Y. Obvious differences between this and the current study are the virus backbone, the host cell used for replication and the use of multiple passages, and these factors may contribute to the discrepancies between the studies. 

Although many differences are seen between previous studies and our use of a non-infectious isolate, an expected difference was the value for the overall mutation frequency. The mutation frequencies are ~3 times higher in TC-83 E3Δ56-59 than in TC-83 that was previously reported [12]. When compared to TC-83 3× [12] and CHIKV C483Y [38] there were ~2–4 times greater mutation frequencies seen in the fidelity mutants in this study. This is likely due to the lack of selection in a non-infectious system and tendency for mutations to be deleterious. The relatively high mutation frequency seen in this study may also be due to a larger proportion of virus genomes being available in the media. Mutations were also incorporated during in vitro transcription [40]. Although these should be equal across all isolates and were seen as common mutations in all isolates, they may inflate the overall diversity because of the limited selection in the non-infectious system. Errors from sequencing can also contribute to the overall mutation frequency and may be mitigated in future assays with the incorporation of molecular barcoding to reduce sequencing errors [41]. However, as the methods used here are similar to previous investigations with VEEV, sequencing errors are unlikely to address discrepancies in the results. 

Viewing mutations by nucleotide positions also highlights similarities and differences between this study and previous reports. Specific mutations were consistently seen across all samples, which has previously been observed [12,42]. However, it is important to note that the sites with increased diversity are different between this study and previously published results. Diversity hotspots in virus genomes are hypothesized to be driven by the host immune response [43,44,45,46]. With the lack of infection from E3Δ56-59 mutants, there are likely minimal selective pressures to shape the viral genome. Therefore, we predict that the consistent mutants seen in the TC-83 E3Δ56-59 isolates do not improve fitness, while mutations seen from previous VEEV experiments may be more advantageous for the virus. The increased number of mutations at the 3′ end of the nsP3 is expected, given the variability in this region across alphaviruses. This region, known as the hypervariable domain, has little conservation between closely related viruses and is able to tolerate mutations [47].

To accurately measure the mutation frequency of specific mutants, we attempted to alter the genome as minimally as possible. This will allow the assessment for fidelity of the replication complex as a whole. It is known that other viral proteins are crucial to virus genome replication, even structural proteins as demonstrated with yellow fever virus [48], so introducing any mutation may have an effect on mutation frequency. While the mutation to create the E3Δ56-59 isolates was minimal, the change will prevent the dissociation of the E2-E3 proteins. Thus, deviations in mutation frequency caused directly by E3Δ56-59 will not be accounted for in this assay.

In conclusion, the use of the E3Δ56-59 mutation in TC-83 has clear limitations based on its lack of infectivity. However, there is a niche to fill in accurately measuring the mutation frequency in potential fidelity mutants that do not have a stable RdRp. The rapid selection of mutations that increase fitness likely leads to the inadvertent measurement of a skewed virus population in passaged samples, even if only one passage is performed. Abolishing infectivity through PE2 cleavage site deletion not only alleviates this issue, but allows virus to be collected from the media, bypassing the necessity for sample enrichment. The PE2 cleavage site is conserved among alphaviruses and has been shown to have similar characteristics in Sindbis virus, Semliki Forest virus and VEEV [25,26,27], so this method is likely useful for other emerging or important alphaviruses such as CHIKV, Mayaro, eastern equine encephalitis, o’nyong’nyong and Ross River viruses. Future research will be performed to examine this. Overall, our method has provided a better understanding of how and to what degree specific mutations alter fidelity, which will be important to aid rational design of live-attenuated vaccines that rely on altered mutation frequency.

## Figures and Tables

**Table 1:** Average frequency of common mutations seen samples. Mutation frequency Analysis A (Analysis B).

Mutation	Gene	TC-83 E3Δ56-59	TC-83 E3Δ56-59 nsP4-G14R	TC-83 E3Δ56-59 nsP4-A96T	TC-83 E3Δ56-59 nsP4-C488Y
C5540A	nsP3	0.0778 (0.1348)	0.1316 (0.2111)	0.1273 (0.2013)	0.1371 (0.2025)
C5555A	nsP3	0.0253 (0.1621)	0.0287 (0.2084)	0.0355 (0.1937)	0.0326 (0.1987)
A7653C	capsid	0.0426 (0.0549)	0.0277 (0.0350)	0.0270 (0.0371)	0.0328 (0.0367)

## Supplementary Materials

The following are available online at https://www.mdpi.com/1999-4915/12/5/546/s1, Figure S1: Mutation frequency of VEEV TC-83 E3 56-59 mutants at coding regions across the genome, Table S1: Average reads per sample for Analysis A, Table S2: Most frequent mutations for each sample for Analysis A.
