# Supplementary material for: Measuring Alphavirus Fidelity Using Non-Infectious Virus Particles
Source: Viruses. 2020 May 15;12(5):546. doi: 10.3390/v12050546 (PMC7291308; doi:10.3390/v12050546)
Supplement: Supplementary file 1 [file viruses-12-00546-s001.pdf]

Supplemental Table 1. Average reads per sample for Analysis A.

Mutant	Replicate	Mean depth positive strand	Mean depth negative strand	Mean depth total
TC-83 E3Δ56-59	1	432.778621	433.5137	866.292321
	2	515.829581	515.142932	1030.97251
	3	399.427836	400.338394	799.76623
TC-83 E3Δ56-59 nsP4-G14R	1	3856.64337	3856.05593	7712.6993
	2	4014.3541	4018.6911	8033.0452
	3	4088.32757	4101.9548	8190.28237
TC-83 E3Δ56-59 nsP4-A96T	1	2276.95314	2276.05515	4553.00829
	2	2234.36763	2238.01117	4472.3788
	3	2678.11353	2686.5644	5364.67792
TC-83 E3Δ56-59 nsP4-C488Y	1	2144.78517	2143.66536	4288.45052
	2	2470.95942	2473.58281	4946.62103
	3	2469.0164	2479.46518	4948.48159

Supplemental table 2. Most frequent mutations for each sample for Analysis A.

Positive strand mutations

VEEV TC-83 E3del56-59 #1

Rank	Mutation	Frequency
1	C5540A	0.0648968
2	A7653C	0.0561224
3	C5553A	0.0343137
4	A580C	0.0338983
5	A780C	0.030303
6	A582C	0.0263158
7	A5719C	0.0254777
8	A1105C	0.0251397
9	A7270C	0.0230263
10	G5528A	0.022792

VEEV TC-83 E3del56-59 #2

Rank	Mutation	Frequency
1	C5540A	0.0946502
2	A7653C	0.035533
3	A7648C	0.0349345
4	A5794G	0.0342679
5	A3990C	0.031746
6	C402G	0.0304569
7	C5555A	0.0294985
8	A2165C	0.0277778
9	A585C	0.025641
10	A6754C	0.0252294

VEEV TC-83 E3del56-59 nsP4-G14R #1

Rank	Mutation	Frequency
1	C5540A	0.136379
2	C401G	0.0429338
3	A2634DEL	0.0339496
4	A7653C	0.0269509
5	C5555A	0.0227137
6	G5528T/A	0.022294
7	A10001C	0.0191424
8	C837A	0.0163872
9	A580C	0.0163366
10	A27C	0.0161744

VEEV TC-83 E3del56-59 nsP4-G14R #2

Rank	Mutation	Frequency
1	C5540A	0.136545
2	C5555A	0.0341928
3	A7653C	0.026936
4	G5528T/A	0.0232843
5	A2634DEL	0.0232558
6	A10001C	0.0197775
7	C837A	0.0192238
8	A780C	0.0163588
9	A418C	0.0163011
10	C5553A	0.015544

VEEV TC-83 E3del56-59 nsP4-A96T #1

Rank	Mutation	Frequency
1	C5540A	0.149942
2	C5555A	0.0441478
3	A2634DEL	0.0382202
4	G5528T/A	0.0296496
5	T11433C	0.0220994
6	A7653C	0.0208034
7	A10001C	0.0184971
8	C11435A	0.0166667
9	A585C	0.0156617
10	C5553A	0.0153302

VEEV TC-83 E3del56-59 nsP4-A96T #2

Rank	Mutation	Frequency
1	C5540A	0.104903
2	C5555A	0.0387755
3	A7653C	0.0303502
4	G5528T/A	0.0236842
5	A585C	0.0192469
6	A7270C	0.0190647
7	A418C	0.0161111
8	A582C	0.0156682
9	A9824C	0.0155039
10	A5199C	0.0147839

VEEV TC-83 E3del56-59 nsP4-C488Y #1

Rank	Mutation	Frequency
1	C5540A	0.152502
2	C5555A	0.04102
3	A2634DEL	0.0352723
4	A7653C	0.0235664
5	C837A	0.02
6	A10001C	0.019802
7	G5528T/A	0.0184226
8	A5199C	0.0143266
9	A9824C	0.0127051
10	A585C	0.0126812

VEEV TC-83 E3del56-59 nsP4-C488Y #2

Rank	Mutation	Frequency
1	C5540A	0.127165
2	C5555A	0.0314524
3	A7653C	0.0310296
4	C837A	0.0223979
5	A10001C	0.0222965
6	A2634DEL	0.0200472
7	G5528T/A	0.0187557
8	A418C	0.017301
9	C5553A	0.0158562
10	A27C	0.0152964

VEEV TC-83 E3del transcript

Rank	Mutation	Frequency
1	C5540A/G/T	0.179036
2	A2634DEL/insT/T/G/C	0.0893827
3	G5528A/T/C	0.0593408
4	C5555A/G/T	0.033924
5	T9957insG/insT/A/C/G/insA	0.0243958
6	T5510A/G/C	0.0236875
7	A7653C/G/T	0.0232678
8	T2507insT/insG/A/C/G	0.0200434
9	C837A/T/G	0.0176365
10	A10001C/G/T	0.0167738

VEEV TC-83 E3del nsP4-G14R transcript

Rank	Mutation	Frequency
1	C5540A/GT/insT	0.180665
2	A2634DEL/insT/G/T/insC/C	0.0450685
3	G5528A/T/C	0.0385345
4	C5555A/G/T	0.0312315
5	T2507insT/insG/A/C/G/insA	0.0309076
6	C837A/T/G	0.0173858
7	T11397insG/A/insT/C	0.0158089
8	T5510A/G/C	0.0155256
9	A7653C/G/T	0.0133255
10	C5553A/G/T	0.0126876

VEEV TC-83 E3del nsP4-A96T transcript

Rank	Mutation	Frequency
1	C5540A/G/T	0.211054
2	A2634DEL/insT/T/G/C	0.086318
3	G5528A/T/C	0.0478007
4	C5555A/G/T	0.0307119
5	T2507insT/insG/A/C/G	0.0295492
6	A7653C/G/T	0.0233614
7	T5510A/G/C	0.019889
8	A418C/G/T/DEL	0.0189843
9	T11397insG/insT/A/C	0.0179663
10	A585C/G/T/DEL/insG/insT	0.0175888

VEEV TC-83 E3del nsP4-C488Y transcript

Rank	Mutation	Frequency
1	C5540A/G/T/insA	0.179951
2	A2634DEL/insT/T/G/C/insA	0.0777254
3	G5528A/T/C	0.039005
4	C5555A/G/T	0.0313867
5	T2507insT/insG/A/C/G/insA	0.0286837
6	T11397/insG/insT/A/C	0.0197568
7	C837A/T/G	0.0173624
8	A7653C/G/T/insC/insG	0.0164076
9	T5510A/G/C	0.0153463
10	C5553A/G/T	0.0145342

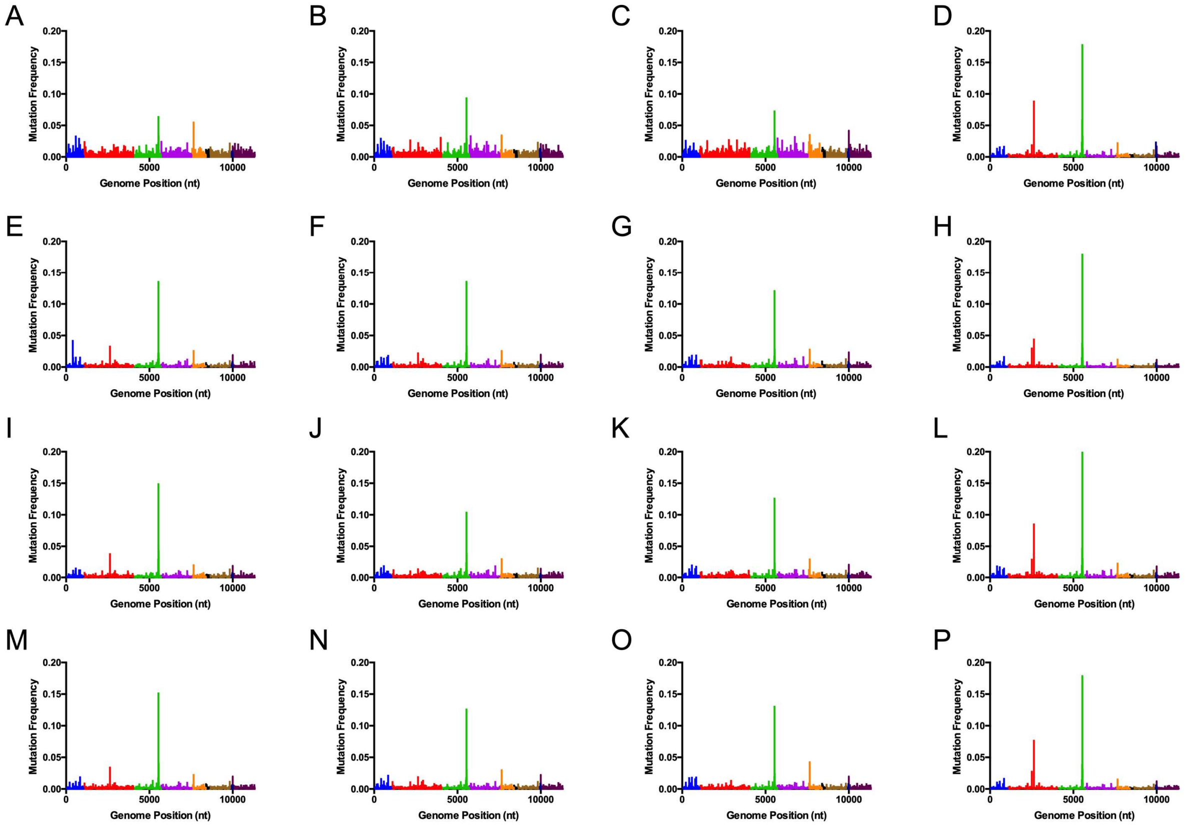

Supplemental Figure 1. Mutation frequency of VEEV TC-83 E3 56-59 mutants at coding regions across the genome. VEEV TC-83 E3 56-59 replicates 1, 2, 3 and transcript(A-D), VEEV TC-83 E3 56-59 nsP4-G14R replicates 1, 2, 3 and transcript (E-H), VEEV TC-83 E3 56-59 nsP4-A96T replicates 1, 2, 3 and transcript (I-L), and VEEV TC-83 E3 56-59 nsP4-C488Y replicates 1, 2, 3 and transcript (M-P). Panels have been analyzed using Analysis A. Graphs show a representative of each specific mutant. Blue=nsP1, red=nsP2, green=nsP3, purple=nsP4, orange=capsid, black=E3, gold=E2, navy blue=6k, maroon=E1.
